# Perceptual influences on self-protective behavior for West Nile virus, A survey in Colorado, USA

**DOI:** 10.1186/s12889-015-1918-8

**Published:** 2015-06-18

**Authors:** Craig W. Trumbo, Raquel Harper

**Affiliations:** Department of Journalism and Technical Communication, Colorado State University, 1785 Campus Mail, Fort Collins, CO 80526 USA; Center for STEM Learning, University of Colorado Boulder, 580 UCB, Boulder, CO 80309 USA

**Keywords:** West Nile virus, Risk Reduction Behavior, Health Communication

## Abstract

**Background:**

The endemic state of West Nile virus (WNv) in North America underscores the need to examine mechanisms influencing human self-protective behavior. Based on previous findings and theory, this study is designed to achieve two specific aims. First, the study will examine self-protective behavior for WNv through a hybridized treatment of the Health Belief Model that includes cognitive, affective, ecological, and proximity risk perception measures. Second, within the resulting hybridized model explore the role of ethnicity in self-protective behavior for WNv.

**Methods:**

Data were collected in Greeley, Colorado, using a self-administered mail survey. 384 completed surveys were returned (49 % completion rate). The questionnaire used items on cognitive-affective risk perception, ecological and proximity risk perception constructs, the Health Belief Model and demographics.

**Results:**

Analysis revealed that newer risk perception models (ecological and proximity) provide some power to explain protective behavior. The psychometric measures of risk perception (cognitive and affective components) provided the best explanatory power. Self-protective behavior was further enhanced by the perception of benefits associated with such actions and the exposure to information cues to action. Hispanic/Latinos demonstrate greater perception of risk/susceptibility and greater exposure to information cues to action, and were more likely to practice self-protective behavior.

**Conclusions:**

The findings in this study point to several useful openings for effective public health communication and intervention for WNv based on affective response, information exposure, and ethnicity. The results also have relevance for vectored diseases generally. It is becoming clear that changes in global climate will bring increased threat from mosquito vectored diseases. Mosquito protection will be an increasingly salient topic for public health communicators in the coming years.

## Background

The endemic state of West Nile virus (WNv) in North America underscores the need to examine the factors that influence personal prevention efforts. Since its appearance in 1999 the epidemiology of WNv in the United States has changed significantly each year both geographically and ecologically. 2003 has thus far been the peak year (9,862 cases, with 264 deaths). Between 2009 and 2011 the total number of cases were less than 1,000 annually, but there has been most recently an increase with 5,674 in 2012 and 2,469 in 2013 [1, 2]. With treatment currently limited to symptomatic care, prevention measures such as integrated mosquito management, insect repellent use, and otherwise avoiding mosquito bites are critical [3–5].

Efforts to engage the public in prevention need further development in order to best motivate the individual behaviors necessary to decrease WNv risk. A better understanding of the manner in which health beliefs and risk perception affect self-protective behavior for WNv will be important to this effort. To better understand the nature of WNv self-protective behavior we called on three theoretical perspectives involving risk perception as well as the Health Belief Model, each of which will be described here briefly.

Several studies examining protective behavior toward WNv have included various measures of risk perception [6–13]. These studies have shown that perception of WNv risk is among the significant predictors of protective behavior, but they have not elaborated how individuals perceive this risk. To improve understanding of how risk perception affects self-protective behavior we first examine the psychometric work by Slovic and colleagues that has demonstrated two dimensions can be seen to describe risk perception: dread and knowledge of the threat [14]. Most recently Slovic and colleagues have been refining this perspective on risk by exploring the role of affect [15, 16]. As a consequence of this and other work on affect, what has emerged is a dual-process risk perception model. Conceptually, this approach follows the form of a model in which indicators are used to tap either cognitive or affective states (e.g., perception of personal control, confidence in scientific knowledge, perception of increasing threat, fear, dread, anxiety).

In addition to this approach to risk perception, we also examine two other perspectives previously reported in the literature on WNv. In two related studies Zielinski-Gutiérrez and Gujral reported findings showing that to understand WNv risk perception it is important to understand individuals’ orientation toward the local ecology in terms of characteristics of the mosquito population [7]. It is also important to understand individuals’ perception of WNv’s physical proximity [11]. This is understood in terms of individual perception of near-by cues such as dead birds and individuals known to have had WNv (those in household, neighborhood). These studies suggest that as the virus spreads into new areas, underlying attitudes toward mosquitoes and the perception of environment and ecology can affect the success of control and prevention measures.

Studies of WNv have also identified a number of other factors affecting protective behavior [6, 8, 10–13, 17, 18]. While sometimes informally used, these factors have been consistent with the Health Belief Model (HBM) with respect to addressing perceived severity and perceived susceptibility to WNv as behavioral predictors. For purposes here, these concepts were more formally incorporated in accordance with the HBM as cues to protective action, barriers to protective action, perceived susceptibility to WNv, and perceived severity of the disease. It is worthwhile to note that the HBM is one of the most robust behavioral models in the domain of prevention [19]. It has been applied across a wide spectrum of topics, including WNv [6, 13].

Finally, this study included an assessment of how Hispanic/Latino ethnicity may affect self-protective behavior in the framework of perceived risk. It is well established that public health communication campaigns should tailor messages and modes of delivery to ethnic subgroups within a target population [20]. In the case of this particular study area a significant Hispanic/Latino population exists and offered an opportunity to include ethnicity as a variable.

Based on previous findings and theory, this study was designed to achieve two specific aims. First, the study examineed self-protective behavior for WNv through a hybridized treatment of the HBM that adds cognitive, affective, ecological, and proximity risk perception measures. Second, within the resulting hybridized model the study explored the role of ethnicity in self-protective behavior for WNv.

This study was based in Greeley Colorado, which is located in Weld County (2003 population 211,879) [21, 22]. In 2003 there was a significant outbreak of WNv in Colorado, with a total of 2,943 human cases and 64 deaths. In Weld County there were 402 total human cases (118 neuroinvasive) and six deaths. Subsequent years prior to this study presented significantly lower levels of infection: eight cases in 2004 (three neuroinvasive), 17 cases in 2005 (four neuroinvasive), 68 in 2006 (16 neuroinvasive) with one death, and 98 in 2007 (18 neuroinvasive). Between 2008 and 2012, the number of cases was less than 131 annually. But in 2013 the number of cases increased to 322. And Colorado continues to have the highest prevalence of WNv in the United States [21].

## Methods

### Participants

While individual protective and public health efforts (especially mosquito abatement) have had an effect, WNv has become endemic to the area under study. Weld County is representative of the counties in the Great Plains that have shown the highest annual and cumulative incidence of WNv infection in the United States [23]. Although populations are fairly small in these primarily agricultural counties, the incidence of neuroinvasive disease in these areas (which often lacks organized mosquito abatement programs) has represented an ongoing public health challenge to local and state authorities. Additionally, Weld County, and especially Greeley, has a significant Hispanic/Latino population (27 %) [22]. The Greeley area’s economy also has a strong agricultural base, with a greater number of outdoor laborers. Therefore, Greeley provides an excellent opportunity for examining ethnic differences of WNv prevention.

Sampling was done by census tracts, with sample lists provided by Survey Sampling, Inc. The community of Greeley has areas of very high and very low Hispanic/Latino populations (with tracts running from about 6 % to 75 %). It was therefore feasible to proportionally sample by ethnicity within census tracts. As the sample areas were all located within a fairly small urban geography they shared a similar degree of background mosquito exposure.

Following approval by the Colorado State University Institutional Review Board, data were collected using a self-administered mail survey. In late fall 2008 the four-page questionnaire (both English and Spanish versions) was mailed to 777 residents proportionally sampled for ethnicity (Hispanic-Latino/Other) in the Greeley, Colorado area. Best practice follow-up (two prompts and a second questionnaire mailing) and $5 cash incentives were employed (in first mailing only).

### Measurement

We employed a set of questionnaire items designed to apply the most recent findings on cognitive-affective modeling of risk perception. Measures capturing ecological and proximity-based conceptualizations of risk were replicated from the previous studies reviewed above. Measures for the HBM were adapted directly from previous studies [24]. Items used in the scale for the dependent variable, self-protective behavior, were taken directly from previous studies and represent a wide range of reasonable actions that individuals might take. Demographic items included age, sex, and Hispanic/Latino ethnicity. Questionnaire items used in this analysis are provided in Table 1.

**Table 1:** Questionnaire items (some items reverse coded)

Protective Behavior (0 never, 1 sometimes, 2 usually, 3 always)
Wore long sleeves/pants to protect against mosquitoes when outside at dawn dusk.
Stayed indoors at dusk or dawn to avoid mosquitoes.
Used mosquito repellant when out at dawn or dusk.
Avoided mosquito-prone areas at dawn or dusk.
Used bug zappers if outdoors at dawn or dusk.
Used citronella candles if out at dawn or dusk.
Wore long sleeves/pants when going outside at dawn or dusk only for a few minutes.
Used mosquito repellant when going outside at dawn or dusk only for a few minutes.
Risk Perception (1 strongly disagree, 2 disagree, 3 neutral, 4 agree, 5 strongly agree)
I do not feel very knowledgeable about the risk of West Nile virus (cognitive)
I don't have any choice over my exposure to West Nile virus. (cognitive)
I believe that the risk of West Nile virus is increasing over time. (cognitive)
The thought of West Nile virus fills me with dread or fear. (affective)
The thought of West Nile virus makes me feel sad or depressed. (affective)
The thought of West Nile virus makes me feel anxious or worried. (affective)
Proximity (1 definitely not, 2 possibly, 3 definitely yes)
I have seen dead birds in the area that might have carried West Nile virus.
Have you ever become sick with West Nile virus?
Has anyone else in your household ever become sick with West Nile virus?
Do you know of anyone in your neighborhood who has become sick with West Nile?
Do you know of anyone outside your neighborhood who has become sick w/ WNV?
Ecology (1 strongly disagree, 2 disagree, 3 neutral, 4 agree, 5 strongly agree)
I don't think about West Nile very often because I almost never see a mosquito
It's too dry in this area of Colorado to have a bad mosquito problem.
My part of town does not have a bad problem with mosquitoes.
Spraying or other city efforts keep mosquitoes under control in my area.
Susceptibility (HBM)(1 strongly disagree, 2 disagree, 3 neutral, 4 agree, 5 strongly agree)
It is likely I will get sick from West Nile virus.
My chances of getting sick from West Nile virus in the next few years are great.
I feel I will get sick from West Nile virus sometime during my life.
Severity (HBM) (1 strongly disagree, 2 disagree, 3 neutral, 4 agree, 5 strongly agree)
I am not worried because most people who get West Nile virus don't get sick.
The thought of West Nile virus scares me.
If I got sick from West Nile virus, the illness would last a long time.
Benefits (HBM) (1 strongly disagree, 2 disagree, 3 neutral, 4 agree, 5 strongly agree)
When I avoid mosquitoes I am doing something to take care of myself.
When I put on repellant I am doing something to take care of myself.
Avoiding mosquitoes will decrease my chances of getting West Nile virus.
Putting on repellant will decrease my chances of getting West Nile virus.
When I avoid mosquitoes I don’t worry as much about West Nile virus.
When I put on repellant I don’t worry as much about West Nile virus.
Using mosquito repellant helps me avoid the bother and discomfort of mosquito bites.
When I use mosquito repellant I set a good example for others.
When I avoid mosquitoes I set a good example for others.
Barriers (HBM) (1 strongly disagree, 2 disagree, 3 neutral, 4 agree, 5 strongly agree)
Avoiding mosquitoes keeps me from doing things I want to do.
Putting on repellant is embarrassing to me.
Avoiding mosquitoes will take too much time.
Putting on repellant will take too much time.
It is hard to remember to put on repellant.
Putting on repellant is unpleasant.
Putting on repellant with DEET is not safe.
Effective mosquito repellant is expensive.
Effective mosquito repellant is difficult to find.
Cues (HBM) (0 none, 1 a little, 2 some, 3 much, 4 very much)
How much information did you see or hear this summer about West Nile virus from these sources:
Newspapers, TV, Radio, Mail, Web, Family, Friends, Doctors

### Data analysis

Aside from the demographic items all measures used in analysis were multi-item scales. Cronbach’s alpha was used to assess scale reliability. Standard descriptive statistics were used to characterize the resulting scales, and Pearson correlations were calculated among the scales. Independent sample *t*-tests were used to compare means and chi-square tests were used to compare proportions. To assess the study’s first aim, a nested multiple regression was run with the self-protective behavior scale (dependent variable) regressed on demographic variables in block 1, the ecology and proximity variables in block 2, the cognitive-affective risk variables in block 3, and the HBM variables in block 4. The blocking strategy was to control all scales for demographics and then allow incremental evaluation of R^2^ for the newer measures, followed by the more tried risk perception measures, and to finally add the best known measures — the HBM. This approach allowed less constrained assessment of the newer measures while also providing comparison of coefficients across models. To assess the second aim the results of the nested regression were re-examined in a stepwise regression of self-protective behavior on the study’s set of theoretical variables. Hispanic/Latino ethnicity was then added as a second block to assess its effect independently.

Post-hoc power analyses were done. The confidence interval for an estimated proportion at *p* < .01 is +/− 5 %. The study has 80 % power to detect a medium effect (*d* = .28, *p* < .05) for a difference in means, 80 % power to detect significance at *p* < .05 for a correlation of *r* = .12, and 80 % power at *p* < .05 to detect a significant R^2^ = .05. Analyses were done using SPSS v.21.

## Results

Data collection was conducted from October 2008 through January 2009 with 384 completed questionnaires returned (49 % return rate). The mean age of the respondents was 55 years, 63 % were female, and 19 % were Hispanic/Latino. Non-Hispanic/Latinos were 78 % while, 1 % black, and 2 % other. Sex and ethnicity were found to be independent (*χ*^2^ = 0.6 *p* = .8), mean age was found not to vary by sex (*t*_(383)_ = 1.2 *p* = .2), and age was found to vary by ethnicity, with Hispanic/Latinos being on average nine years younger than others (*t*_(383)_ = 5.5 *p* < .01). Comparisons were made between the sample and population figures for Greeley, Colorado from the U.S. Census (2013 American Community Survey estimates). The sample is older than the population (population = 31 years, *t*_(383)_ = 29.6 *p* < .01), more female (population = 51 % female, *t*_(383)_ = 4.5 *p* < .01), and less Hispanic/Latino (population = 27 %, *t*_(383)_ = 3.8 *p* < .01).

Additive scales were created for each of the risk perception measures. Table 2 reports the characteristics of these scales as well as the scales for protective behavior and the HBM components. A correlation matrix was created for the scale variables. Table 2 also reports these values. Of greatest interest are the correlations in the bottom right corner of the matrix, among the four risk perception constructs investigated in this study. While the affective and cognitive components of the risk perception measures were positively correlated with each other, their correlations with ecological perception were opposite in valence. Perceiving risk based on cognitive factors is positively associated with the ecological variable indicating perception of greater risk. That relationship is logical as ecological risk perception is based on an understanding of the environment. However, perceiving risk based on affective factors was negatively associated, indicating that those who perceive greater risk on the affective dimension feel that WNv is not a problem based on mosquito ecology.

**Table 2:** Descriptive statistics and Pearson correlations for scale measures (N = 384)

	M	SD	α	1	2	3	4	5	6	7	8	9
1. Protect	7.6	4.2	.73	.30^**^	-.14^**^	.31^**^	.34^**^	.30^**^	-.12^*^	.30^**^	.16^**^	.15^**^
2. Benefit	35.1	4.83	.82	1	-.21^**^	.11^*^	.14^**^	.25^**^	-.21^**^	.18^**^	.15^**^	.04
3. Barriers	22.36	5.86	.82		1	-.03	.06	-.02	.25^**^	.16^**^	-.10	.17^**^
4. Cues	7.35	4.81	.76			1	.30^**^	.19^**^	-.04	.20^**^	.12^*^	.22^**^
5. Suscept	8.83	2.60	.81				1	.48^**^	.06	.41^**^	.29^**^	.29^**^
6. Severity	10.46	2.33	.78					1	-.01	.51^**^	.34^**^	.17^**^
7. Risk Cognitive	5.16	1.66	.45						1	.16^**^	-.19^**^	-.10
8. Risk Affect	7.85	2.92	.88							1	.32^**^	.14^**^
9. Risk Ecology	8.58	3.01	.75								1	.24^**^
10. Risk Proximity	7.46	1.99	.55									1

*p < .05 **p < .01

Turning to proximity, we see that while the cognitive-proximity correlations were not significant, the affect-proximity associations were. Here, having a greater level of proximal exposure to WNv phenomena (seeing dead birds, sick people, etc.) is linked to greater affective risk perception (e.g., fear, anxiety). Finally, having a greater perception of WNv proximity risk is associated with a greater perception of ecological risk. It is also worth noting that the elements of the HBM were significantly associated in 15 of the 20 correlations with the risk perception variables. The relative lack of associations between the HBM variables and cognitive risk perception is interesting, as is the strength of these associations with affective risk perception. Finally, all model variables were significantly correlated with self-protective behavior.

The results of the nested regression model assessing for first aim are presented in the top of Table 3. Within the block of demographic variables, the results show that age and sex are both initially significant. Older respondents and females are more likely to practice self-protective behaviors. The demographic variables remain significant in the second block as the ecological and proximity risk variables are added, which improve the model significantly.

**Table 3:** Nested and Stepwise Regressions on Self-Protective Behavior (N = 384)

Model	IVs	B	SE	β	*t*	*p*	ΔR^2^	*F*_*(df1,2)*_	*p*
1	age	.04	.01	.15	2.95	>.01			
	sex ^a^	-1.21	.43	-.14	-2.82	>.01	.03	5.9_(2,381)_	>.01
2	age	.05	.01	.19	3.67	>.001			
	sex	-1.16	.42	-.13	-2.72	>.01			
	ecology	.15	.07	.11	2.10	>.05			
	proximity	.31	.10	.15	2.97	>.01	.05	9.3_(2,379)_	>.001
3	age	.04	.01	.14	2.76	>.01			
	sex	-.71	.42	-.08	-1.68	.093			
	ecology	.01	.07	.01	0.13	.890			
	proximity	.25	.10	.12	2.38	>.05			
	risk cog.	-.41	.17	-.16	-3.21	>.001			
	risk affect	.36	.07	.25	4.63	>.001	.07	14.9_(2,377)_	>.001
4	age	.03	.01	.13	2.76	>.01			
	sex	-.74	.39	-.09	-1.88	.060			
	ecology	.05	.07	.04	0.68	.491			
	proximity	.12	.10	.06	1.20	.231			
	risk cog.	-.23	.12	-.09	-1.89	.058			
	risk affect	.18	.08	.13	2.19	>.05			
	suscept	.28	.08	.18	3.24	>.001			
	severity	.07	.10	.04	0.74	.458			
	benefit	.15	.04	.18	3.75	>.001			
	barriers	-.06	.03	-.09	-1.75	.081			
	cues	.16	.04	.18	3.74	>.001	.14	14.5_(5,372)_	>.001
							.28	13.3_(11,372)_	>.001
Stepwise (forced entry of Ethnicity)									
1	age	.04	.01	.15	3.27	>.01			
	risk affect	.16	.07	.12	2.27	>.05			
	risk cog.	-.27	.12	-.11	-2.37	>.05			
	suscept	.31	.08	.19	3.90	>.001			
	benefit	.17	.04	.20	4.30	>.001			
	cues	.17	.04	.19	4.09	>.001	.27	18.3_(8,375)_	>.001
2	ethnicity ^b^	1.04	.52	.10	1.99	>.05	.01	4.0_(1,375)_	>.05
							.28	18.3_(8,375)_	>.001

*0* = other

^a^1 = male, 0 = female ^b^ 1 = Hispanic/Latino

The cognitive and affective risk components improve overall model fit significantly and also control the effect of both sex and ecological risk. Perception of greater risk based on affective aspects is associated with greater use of protective measures, while risk perception based on cognitive aspects has the opposite association. Within the block for the HBM, perceiving greater benefits to self-protective behaviors, feeling more susceptible to WNv, and having been exposed to more cues to action predicted self-protective behavior and improved model fit by 14 %. The HBM measures control the effect of proximity and cognitive risk while affective risk perception remains significant in this last block.

The results of the stepwise regression model assessing the second aim are presented in the bottom of Table 3. Here we see the results of the nested model replicated more efficiently. In the second block Hispanic/Latino ethnicity improves the model significantly, but with a modest effect size. To further support the second aim of the study the set of predictor variables were compared by ethnicity. Several of the variables had non-significant differences: cognitive risk perception, perceived severity, perceived benefits, and risk proximity. There were, however, significant differences for Hispanic/Latinos on the remaining variables. As a group, they were more likely to practice self-protective behavior (M = 9.2 vs. 7.2, *t*_(383)_ = 3.6 *p* < .01), present a stronger affective risk response (M = 9.0 vs. 7.6, *t*_(383)_ =3.9 *p* < .01), perceive greater susceptibility (M = 9.7 vs. 8.6, *t*_(383)_ = 3.5 *p* < .01), report fewer barriers (M = 19.8 vs. 22.9, *t*_(383)_ = 4.1 *p* < .01), perceive less ecologically-based risk (M = 7.3 vs. 8.9, *t*_(383)_ = 4.3 *p* < .01), and report exposure to more information (M = 9.4 vs. 6.7, *t*_(383)_ = 4.2 *p* < .01). The last variable, cues, was unpackaged to see what sources might be most relevant. Respondents overall reported greater exposure to mediated cues (tv, newspaper, radio) compared to personal cues (family, friends, doctors)(media M = 3.7, personal M = 2.9, *t*_(383)_ = 5.9 *p* < .01) The analysis of ethnicity found that Hispanic/Latinos were exposed to significantly more messages via television (M = 1.8. vs. 1.4, *t*_(383)_ = 3.2 *p* < .01), family (M = 1.6. vs. 1.0, *t*_(383)_ = 3.6 *p* < .01), and doctors (M = 1.1 vs. 0.4, *t*_(383)_ = 5.8 *p* < .01).

## Discussion

### Study aims

The first aim of this study was to predict self-protectiuve behvior using the HBM along with an additional set of risk measures. Of the three aditional perspectives examined, the measures based on the cognitive-affective risk percetpion model performed best, with the affective component being clearly superior (dread, anxiety, worry). It is not clear why the cognitive component of this measure presented weaker or directionally opposite results. It seems fairly clear that the affective measures were more effective as a group (certainly more reliable). The three items developed for the cognitive aspect of the measure (knowledge, control, increasing risk) were more difficult to construct, as the cognitive aspects of risk perception that make sense in the context of a technology (their traditional contextual application) did not lend themselves as readily to the risk associated with a vector-borne infectious disease. The inclusion of the item on whether or not WNv is seen to be increasing in prevalence may have also tapped more of an affective response.

The success of the other two risk perception constructs is more provisional. While the ecological measure does have some utility, it was not sufficiently robust to remain in the model alongside the other measures. Further analysis of this measure is certainly warranted to examine its potential use with some more discrete aspects of the data set. Its bivariate relationships with susceptibility and severity suggest it may be useful in other approaches using the HBM. Also, casual examination of other aspects of ecology suggested interesting avenues for future analysis. For example, there was a significant difference in means across ethnicity on the ecology score, with Hispanic/Latinos showing a greater risk response based on the ecological factors.

The second aim of the study was to specifically examine the significance of ethnicity within a trimmed, hybrid model. While this is a single study with a limited sample, the findings do support previous work on WNv showing differential response by Hispanic/Latino persons. Importantly, we see that Hispanic/Latinos were more likely to practice self-protective behavior. This is likely especially motivated by their greater perception of risk/susceptibility and greater exposure to information cues to action.

### Implications for practice

One of the important aspects of these findings for public health practitioners resides in the role of affect in individual behavior. While an understanding of the ecological dimension of WNv and direct experience with risk indicators such as dead birds can play a role in shaping public response, the strongest motivator for protective behavior remains an emotional response to the threat. This may suggest that public health communications on WNv may not benefit as strongly from fact-based messages as they might from more emotional treatment, perhaps especially through the use of personalized narratives.

The three significant variables from the HBM also offer further insight into potential approaches for public health practice. The perception of individual susceptibility points to an opening to gain the public’s attention on the importance of avoiding mosquitoes during a WNv outbreak, and the influence of perceived benefits suggests a reinforcing dimension to that line of persuasion: you are susceptible and you will benefit from these actions. Underscoring this most importantly is the final inclusion of information effects, cues to action, that show that messaging on this topic may influence behavior, although the causal ordering cannot be empirically supported here.

Differences on ethnicity are also important. The differences seen in specific information sources reported by Hispanic/Latinos illustrates some possibilities for practice. Greater exposure to television messages may suggest a higher level of attentiveness to the topic. More exposure to information from doctors may suggest either an outreach effort by physicians to inform a more vulnerable population, or more exposure to such information due to a greater likelihood of needing to seek diagnosis or treatment. The stronger role of family members may be based in the cultural domain. Taken together, these findings on ethnicity point toward potentially important avenues for public health message tailoring.

Given the sometimes controversial nature of mosquito control and news media treatment of health risks (and WNv in particular) it is worth mentioning the topic of media effects. As reported above, respondents indicated greater exposure to the three mediated cues as compared to the three personal cues. It is tempting to speculate that some aspects of attitude and behavior might be affected by intense or “hyped” media reporting. However, we did not monitor the media environment prior to or during the study period and do not have specific media exposure measures that would allow us to examine such a direct effect. It’s also well established that media don’t affect attitudes or behaviors in a direct fashion, but rather interact with and flow through interpersonal sources. Our study was simply not designed to examine this phenomenon.

In a broader sense it is also worthwhile to conclude with a perspective on mosquito-vectored diseases generally, not only WNv. It is becoming clear that changes in global climate will bring increased threat from mosquito vectored diseases. This is anticipated for WNv as well as other diseases, most prominently dengue and Chikungunya virus as the Asian tiger mosquito expands its range into North America [25, 26]. Mosquito protection will be an increasingly salient topic for public health communicators in the coming years.

### Limitations

The measures used for the three exploratory scales, while based on extant literature, were assembled ad hoc. More rigorous item development through focus groups and survey pre-testing would have certainly yielded superior measures. This may be especially of concern for the scales measuring cognitive risk perception and proximity risk perception, as the reliabilities were low. The sampling strategy was successful in providing a more representative capture of ethnicity that likely would have been the case for a non-proportional approach. While the survey response rate of 49 % is generally considered acceptable for self-administered mail surveys it does nonetheless represent a limitation, especially as significant differences were found between sample values and Census figures. This may be a notable hindrance in further analyses of these data focusing on ethnicity, as the data only contain 74 cases for the Hispanic/Latino segment. Finally, the study design precludes the basis for causal interpretations.

## Conclusions

The findings in this study point to several useful openings for effective public health communication and intervention for WNv based on affective response, information exposure, and ethnicity. The results also have relevance for vectored diseases generally. It is becoming clear that changes in global climate will bring increased threat from mosquito vectored diseases. Mosquito protection will be an increasingly salient topic for public health communicators in the coming years.
